# The effect of 12 weeks of basic soccer training on violence tendency, psychological resilience, social anxiety in 12–14 years old children

**DOI:** 10.3389/fpsyg.2025.1693298

**Published:** 2025-10-30

**Authors:** Büşra Özcan, Baha Engin Çelikel, Ramazan Erdoǧan, Mustafa Karadaǧ, Volkan Aydoǧdu, Fatih Mehmet Uǧurlu, Eyüp Bozkurt, Mehmet Turan, Meryem Koçal, Serdar Orhan

**Affiliations:** 1 School of Physical Education and Sports, Siirt University, Siirt, Türkiye; 2 Faculty of Sport Sciences, Firat University, Elazig, Türkiye; 3 Faculty of Sport Sciences, Munzur University, Tunceli, Türkiye; 4 Faculty of Sport Sciences, Bitlis Eren University, Bitlis, Türkiye; 5 Faculty of Education, Firat University, Elazig, Türkiye; 6 Faculty of Education, Hatay Mustafa Kemal University, Hatay, Türkiye; 7 Kuşadasi Sports Football Club, Aydin, Türkiye

**Keywords:** violence tendency, psychological resilience, social anxiety, footbal, sports psychology

## Abstract

**Background:**

Regular exercise during childhood impacts not only physical development but also emotional stability, social adaptation, and behavioral control. Therefore, the present study aimed to examine the effects of a 12-week introductory football training programme on violent tendencies, psychological resilience, and social appearance anxiety in children aged 12–14.

**Methods:**

A randomized controlled design was used, with 40 children assigned to an experimental group (*n* = 20) or a control group (*n* = 20). The experimental group completed 60-min football sessions three times a week, while the control group did not participate in structured activity. The program included a 20-min warm-up, technical and tactical exercises, game-based activities, and a cool-down, targeting physical, technical, tactical, and psychosocial development. Data were collected using the VTS, PRS, and SAAS scales.Statistical analyses were performed using SPSS version 25 (IBM Corp., Armonk, NY, USA), with mixed-design ANOVA, significance set at *p* < 0.05, and effect sizes reported as partial eta-squared (ηp^2^).

**Results:**

No significant difference was observed in violence tendency scores in the experimental group (*p* = 0.939). However, a decrease was observed in psychological resilience scores in comparison to pre-test values, and this decrease approached the threshold of significance with a medium effect size (*p* = 0.153, ηp^2^ = 0.053). This finding suggests that the intervention may have a limited effect on psychological resilience. While no significant change was detected in the social appearance anxiety variable in the experimental group (*p* = 0.120), a significant decrease in anxiety levels was observed in the control group (*p* = 0.029). This finding suggests that factors other than the experimental intervention may have been effective in the control group.

**Conclusions:**

In conclusion, the 12-week football training programme demonstrated no significant short-term effect on violent tendencies or social appearance concerns, but may have a temporary effect on psychological resilience. The findings indicate that the impact of sports participation on psychosocial development in children is multifaceted and contingent on individual differences.

## 1 Introduction

It is acknowledged that spectating sports exerts considerable influence on the societal well-being of the public, encompassing physical, mental, and social aspects (Yuhei et al., 2015). This study undertakes an examination of theoretical conceptualisations of interest in vicarious sporting events. To elaborate further, the present study focuses on the manner in which participation in sporting activities influences self-esteem, a pivotal variable in any individual's daily psychological functioning and self-regulation. This influence exerts significant consequences for health and interpersonal relationships (Zeigler-Hill, 2013). As demonstrated in the relevant literature, studies conducted hitherto have examined the relationship between sport and self-esteem (Bizman and Yinon, 2002; Hirt et al., 1992). However, these studies obtained their data solely from post-game measurements.

Football is one of the most widely played and watched sports worldwide, attracting diverse segments of society. Its dynamic structure and emotional intensity—including joy, excitement, sadness, uncertainty, and competition—contribute significantly to its global popularity (Kuper, 1996). Beyond entertainment, football provides psychological and social satisfaction, with fans often developing strong emotional connections to their teams (Sezer and Çelikel, 2021). In some cases, this intense identification can lead individuals to perceive match outcomes as highly significant, generating extreme emotional responses depending on the results (Bilir and Sangün, 2014). Supporters experience a shared range of emotions, feeling happiness when their team wins and sorrow when it loses, while simultaneously embodying pride, anger, and frustration (Talimciler, 2006).

Although violence is a persistent social issue, its manifestation in sports varies (Guilbert, 2004). Football has historically witnessed violent incidents influenced by match dynamics, spectator behavior, and environmental conditions (Kayaoglu, 2004). The mid-20th century saw a notable increase in football-related violence in England, which subsequently spread to other countries, causing both physical and psychological harm to athletes and spectators (Eric, 1999; Sezer, 2006). Similar trends have been observed in Turkey, where violent incidents during football matches have resulted in fatalities and injuries, highlighting the influence of group dynamics in open sports environments on individual behavior (Erkal et al., 1998). All participants in sports—including athletes, coaches, and spectators—are expected to interact within a framework of respect and sportsmanship (Narto, 2024). Nevertheless, aggression and violent behaviors continue to occur in many sports contexts (Düzce, 2019). Violence arises from a combination of individual, environmental, and societal factors, often causing physical, emotional, and psychological harm (Toksöz, 2022). The World Health Organization defines violence as behaviors intended to intimidate individuals or groups, causing psychological harm, hindering socialization, inflicting injury, or potentially leading to death (Dünya Saglik Örgütü, 1996). Biçer (2022) emphasizes that violent environments are characterized by the absence of peace, compromised individual rights, pervasive fear, and behaviors that physically or emotionally harm individuals.

In addition to external violence, athletes frequently encounter various forms of stress in sports settings (Guilbert, 2004). Psychological resilience, defined as the ability to cope with and recover from adversity, plays a crucial role in managing these pressures (Basim and Çetin, 2011). Resilience encompasses both personality traits and adaptive behaviors influenced by environmental conditions (Karademir and Açak, 2019). Developing resilience is particularly important for young athletes, as it enables them to endure the mental and emotional demands of competitive sports (Fletcher and Sarkar, 2012).

Social anxiety, another critical factor in youth sports, is defined as fear of negative evaluation in the presence of others (Kashdan, 2007). It can impair task execution, reduce communication effectiveness, and limit social interactions, preventing individuals from achieving optimal performance (Dayhoff, 2000; Kashdan, 2007; Kaya et al., 2012; Gümüş, 2006). In performance-based sports, social anxiety may restrict athletes' ability to demonstrate full potential, affecting both personal development and team outcomes (Ildefonso et al., 2017).

### 1.1 Importance of the study

Early adolescence (ages 12–14) is a critical period for social, emotional, and behavioral development (Pfeifer and Allen, 2021). Participation in structured sports programs such as football can have a significant impact on psychosocial outcomes, including reducing violent tendencies, enhancing psychological resilience, and mitigating social anxiety. However, empirical research examining these effects in young football players remains limited. Understanding these relationships is essential for developing interventions and training programs that foster both mental and social well-being in youth.

### 1.2 Research aim and hypotheses

The primary aim of this study is to investigate the effects of 12-week basic football training on violence tendencies, social anxiety, and psychological resilience in 12–14-year-old children. The study is guided by the following hypotheses:

Participation in regular football training reduces violence tendencies among young players.

Football training positively influences psychological resilience in children.

Engagement in football decreases social anxiety levels in young athletes.

By addressing these hypotheses, the study seeks to provide evidence on how structured sports programs can contribute to the psychosocial development of children and support the cultivation of mentally and socially healthy individuals.

## 2 Materials and methods

### 2.1 Research model

This study examined the effects of football participation on violence tendency, psychological resilience, and social appearance anxiety in children aged 12–14 using an experimental research design. Experimental research is commonly employed to determine causal relationships by manipulating independent variables and observing their effects on dependent variables under controlled conditions (Büyüköztürk et al., 2012). This method allows researchers to assess the impact of a structured football training program on the psychosocial characteristics of young individuals by comparing pre-test and post-test measurements between experimental and control groups.

### 2.2 Research group

The effect size of the study was examined using G-power analysis. The determination of the requisite number of participants was conducted utilizing the GPower 3.1 software. In a two-sided test, the minimum detectable effect sizes for α = 0.05 were calculated as dz ≈ 0.54 (power = 0.80), dz ≈ 0.62 (power = 0.90), and dz ≈ 0.69 (power = 0.95). The study sample consisted of 40 children aged 12–14, who were selected through purposive sampling. The study was designed as a randomized controlled experimental trial. Participants were randomly assigned to two groups: the Exercise Group (E.G) and the Control Group (C.G). The exercise group was defined as n=20, and the control group as *n* = 20.

The subjects assigned to the exercise group underwent a 12-week exercise regime. The control group did not engage in any sporting activities. This study was conducted between June and August in 2024. The football players were provided with detailed information about the study. Participation was entirely voluntary, and data were collected via structured questionnaires administered face-to-face during training sessions. Prior to data collection, participants and their legal guardians were informed about the study objectives, procedures, confidentiality measures, and voluntary participation. Individuals who did not consent were excluded from the study. The sample comprised children with varying levels of football experience, thus enabling the researchers to examine the potential moderating effects of sport experience on the measured outcomes. The determination of the inclusion group was achieved through the random assignment of subjects in the sample to two groups, utilizing a computerized programme (https://www.randomizer.org/). Participants with chronic or other medical conditions were excluded from the study. Verbal and written informed consent was obtained from the parents of all participants before starting the study. Although this study did not follow any specific guidelines for clinical research, it was conducted within the framework of the ethical principles set forth in the Declaration of Helsinki and the research standards applicable in the social sciences.

**Inclusion criteria:**

Were aged between 12 and 14 years.

Participated voluntarily and provided informed consent along with parental/guardian approval.

Attended at least 80% of the 12-week football training sessions.

### 2.3 Data collection procedure

Before administering the questionnaires, participants received detailed verbal explanations regarding the study's purpose, the voluntary nature of participation, and the confidentiality of their responses. Surveys were designed to maintain participant anonymity, with no personally identifiable information collected. Data were collected using a before-after approach, providing a snapshot of participants' psychosocial status before and after the football training intervention. The training program itself was structured to provide consistent physical activity while fostering teamwork and social interaction, which may influence the psychosocial variables under study.

### 2.4 Data collection instruments

#### 2.4.1 Violence Tendency Scale in Team Sports

The Violence Tendency Scale in Team Sports (VTS) was employed to evaluate participants' predisposition toward violent behaviors in team sports contexts. This 5-point Likert-type scale consists of 17 items divided into three sub-dimensions: Physical Violence (items 1–7), Verbal Violence (items 8–13), and Emotional Violence (items 14–17). Participants indicate the extent to which each statement reflects their own behavior, with responses ranging from 1 (Strongly Disagree) to 5 (Strongly Agree). Higher total scores indicate a higher propensity for violence, while lower scores indicate lower predisposition. The internal consistency reliability coefficients were 0.82 for Physical Violence, 0.80 for Verbal Violence, and 0.73 for Emotional Violence, demonstrating satisfactory reliability for research purposes (Aslan and Ugraş, 2021). This scale is widely used in sports psychology research to identify behavioral tendencies in team settings and has shown consistent psychometric properties in adolescent populations (Haskan and Yildirim, 2012).

#### 2.4.2 Psychological Resilience Scale

The Psychological Resilience Scale (PRS) was used to assess participants' capacity to cope with stress and adversity. This single-factor, Likert-type scale includes 12 items, with responses ranging from 1 (Not at all appropriate) to 5 (Completely appropriate). Total scores range from 12 to 60, with higher scores reflecting higher levels of psychological resilience. The scale does not include reverse-coded items. Reliability analyses demonstrated a Cronbach's alpha of 0.91, and factor loadings ranged from 0.39 to 0.88, confirming both internal consistency and construct validity (Arslan, 2015). The PRS has been validated for use in youth populations, making it suitable for assessing resilience in children participating in sports programs (Van Der Meer et al., 2018).

#### 2.4.3 Social Appearance Anxiety Scale

The Social Appearance Anxiety Scale (SAAS) was employed to measure participants' concerns regarding their physical appearance in social contexts. The scale consists of 16 items scored on a 1–5 Likert-type scale, with total scores ranging from 16 to 80. Low anxiety is defined as 16–37, moderate anxiety as 38–59 and high anxiety as 60–80. Higher scores indicate greater social appearance anxiety, whereas lower scores reflect reduced anxiety. Item 1 is reverse-coded to control for response bias. The internal consistency reliability coefficient of the scale is 0.84, indicating good reliability for assessing social appearance anxiety among youth (Hart et al., 1989).

### 2.5 Weekly traning program

The 12-week football training program is structured to support the physical, technical, tactical, and psychosocial development of young players aged 12–14. Three training sessions each week (Monday, Wednesday, and Friday) include a 20-min warm-up, technical exercises, game-based practices, and a cool-down. Physical activities such as running, changes of direction, and speed drills develop endurance, agility, and acceleration and deceleration skills, while dribbling, shooting, passing, and body control exercises enhance technical proficiency. Team games, offensive and defensive drills, and tournaments strengthen players' tactical awareness, decision-making, and cooperation skills. Coordination and rhythm exercises support neuromuscular control, while flexibility and dynamic movements help prevent injuries. The program is structured according to the principles of progressive loading and progression, offering a balanced program that begins with fundamental skills and covers developmental areas such as tactical coordination and coordination under game stress. Overall, this program supports both physical and technical skills and psychosocial outcomes such as motivation, endurance, and social interaction.

### 2.6 Data analysis

The data collected in this study were analyzed using the Statistical Package for the Social Sciences (SPSS) version 21.0. Prior to the main analysis, all datasets underwent a comprehensive screening process to ensure accuracy, completeness, and reliability. This included checks for missing values, outliers, and inconsistent or implausible responses. Any participant failing to meet inclusion criteria or exhibiting incomplete responses was excluded from the dataset.

**Participants were excluded if they:**

Did not provide consent or withdrew from the study.

Missed more than 20% of training sessions.

Had chronic illnesses, recent injuries, or conditions preventing full participation in football activities.

Submitted incomplete or inconsistent survey responses.

After applying these criteria, a total of 40 participants were included in the final analysis, ensuring a homogeneous sample in terms of participation and exposure to the training program.

Statistical Analysis All analyses were performed bilaterally (95% confidence level) and at a significance level of α = 0.05. The mean value and standard deviation of the data are presented in the form of descriptive statistics. The following section will address the subject of data controls. Outliers were defined as |z|>3; those that were biologically plausible were retained, and sensitivity analysis was performed when necessary. The following data is absent: Participants with mismatched pre-test/post-test results were excluded from the list and re-analyzed using a mixed model for verification purposes. The concepts of normality and variance homogeneity are examined. Data were analyzed using the Shapiro–Wilk and Levene tests. Analyses revealed that the data were normally distributed and the variances were homogeneous. Therefore, parametric tests were applied in the analyses. A 2 (group: EG vs. CG) × 2 (time: Pre vs. Post) mixed ANOVA was performed for each marker. In instances where the interaction was deemed to be of significance, the simple effects were subjected to rigorous examination through the utilization of the Bonferroni correction method. Interaction: The F, *p*-value (P3) and partial η^2^ (np^2^) were reported. Within-group change: The paired t-test or, if deemed inappropriate, the Wilcoxon test (P1) was utilized. The percentage change was calculated using the formula: [(Post – Pre)/Pre] × 100. Effect sizes: The results of this study will be presented with Cohen's d or Hedges' g, alongside their 95% confidence interval. Further analyses are required. Prior to the implementation of the experiment, the equality of the two groups was established through the utilization of an independent t-test. In instances where the assumptions were not met, robust ANOVA or non-parametric methods were applied.

#### 2.6.1 Effect size and confidence intervals

In the two-tailed test, the minimum detectable effect sizes for α = 0.05 were calculated as dz ≈ 0.54 (power = 0.80), dz ≈ 0.62 (power = 0.90) and dz ≈ 0.69 (power = 0.95). Ninety-five percent (95%) confidence intervals were calculated for mean differences to indicate the precision and reliability of the estimates (Cohen, 2013).

#### 2.6.2 Data visualization and interpretation

Results were presented using tables and graphs to visually illustrate trends, differences, and variability among the variables. These visualizations facilitated interpretation of the intervention's effects on psychosocial outcomes and supported a comprehensive understanding of the data.

#### 2.6.3 Ethical and procedural considerations in data analysis

All analyses were performed on anonymized datasets to maintain participant confidentiality. Data handling followed ethical guidelines, ensuring that results were unbiased and reflective of the true effects of the football training program.

### 2.7 Research ethics

Ethical approval was obtained from the Ethics Committee of Siirt University under decision number 2023/6048, dated December 14, 2023.

## 3 Results

The mean age of the experimental group was 13.2 ± 0.6 years, mean height was 156.4 ± 6.1 cm, and mean body weight was 46.5 ± 6.3 kg. The mean age of the control group was 13.0 ± 0.7 years, mean height was 154.2 ± 5.8 cm, and mean body weight was 48.1 ± 5.9 kg. The findings show that there is no substantial difference between the groups in terms of age, height and body weight, and the groups are homogeneous in terms of these variables.

**Figure 1:**
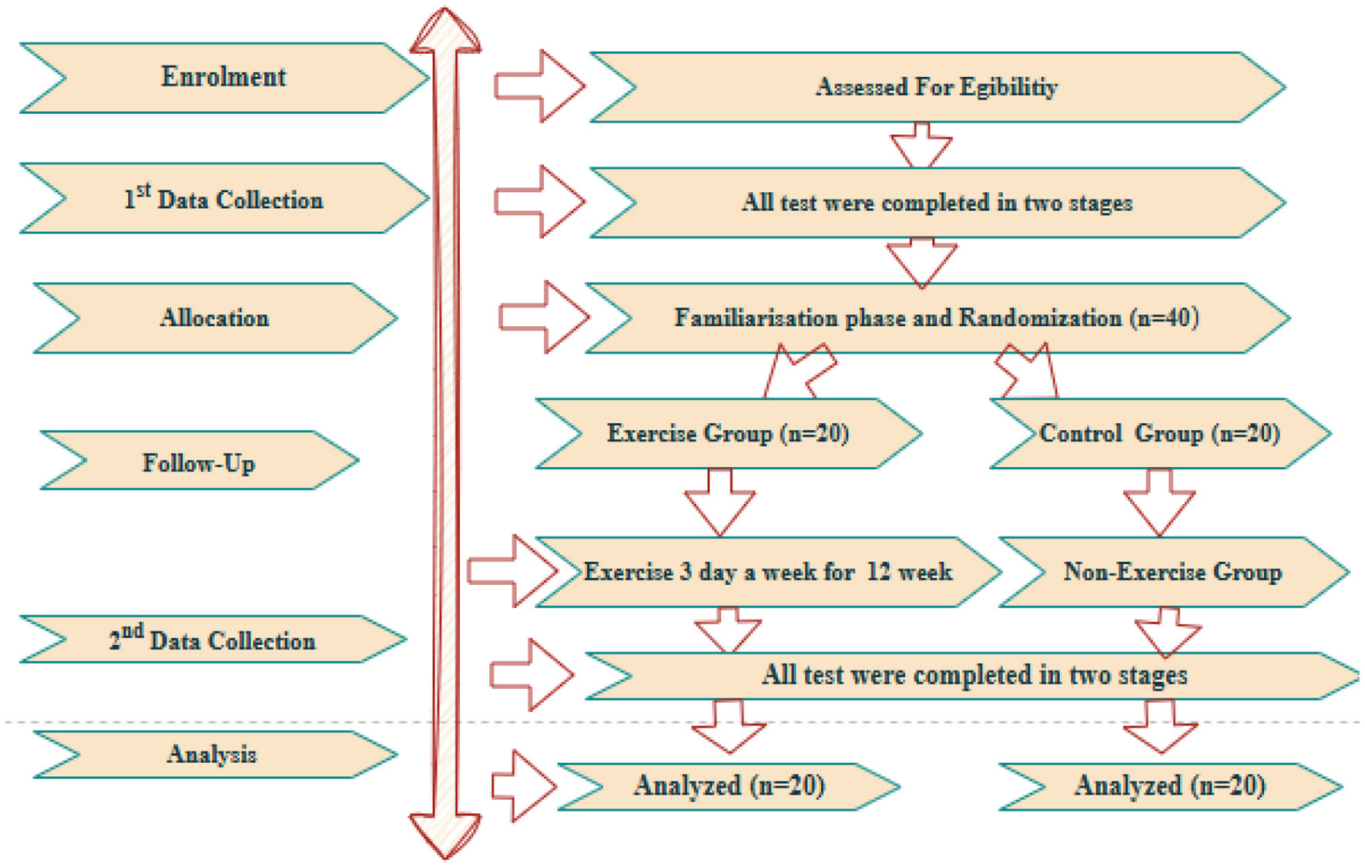
Experimental design.

**Figure 2:**
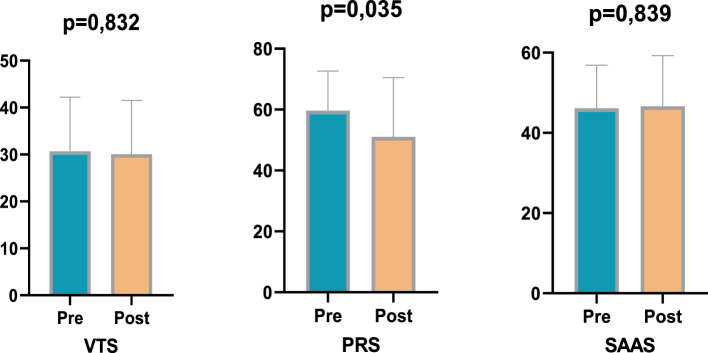
Pre- post test comparisons of the exercise group.

**Figure 3:**
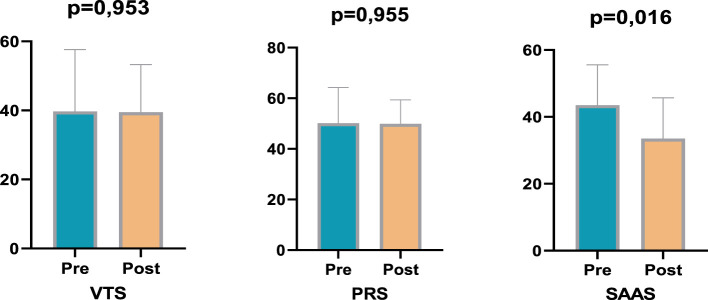
Pre- post test comparisons of the control group.

The findings of the study demonstrate divergent trends in the measurements of the exercise and control groups prior to and following the training period (Figure 1). An analysis of the Violence Tendency Scale (VTS) results reveals that the pre-test average in the exercise group was 30.70, which decreased to 30.05 in the post-test (Table 1). In a similar vein, within the control group, the mean score prior to the intervention was 39.70, decreasing to 39.45 following the post-intervention evaluation. The decreases observed in both groups were found to be statistically insignificant (*p* > 0.05). Moreover, no significant discrepancy was identified in the comparisons between the groups (*p* < 0.939). This finding indicates that the exercise programme or the time effect did not cause a significant change in violent tendencies. With regard to the Psychological Resilience Scale (PRS), a significant decrease was observed in the exercise group (Figure 2). The mean score, which was 59.65 in the pre-test, decreased to 51.00 in the post-test. This change is thought to be due to training intensity (*p* < 0.05). Training intensity appears to affect the individual. This modification approached statistical significance and demonstrated a medium effect size (*p* = 0.035; ηp^2^ = 0.053). In the control group, the pre-test average of 50.15 decreased to 49.90 in the post-test, but this change was not found to be significant (*p* = 0.955). This finding indicated a decrease in the subjective perception of improvement in subjects in the exercise group, while the control group demonstrated consistent values (Table 2). The findings of the Sports Anxiety and Aggression Scale (SAAS) indicate a notable difference between the groups (Figure 3). In the exercise group, the pre-test score was 46.10, rising to 46.65 in the post-test. However, this increase was not statistically significant (*p* > 0.05). In the control group, the mean pre-test score of 43.50 decreased to 33.50 in the post-test, and this change was found to be statistically significant (*p* < 0.05; ηp^2^ = 0.120). Similarly, the significant decrease in social appearance anxiety (SAAS) observed in the control group suggests that uncontrolled exogenous variables, such as school environment, family dynamics, peer relationships, or other contextual factors, may have been attributable to the experimental intervention alone. This constitutes a significant limitation in terms of the study's internal validity, as unanticipated changes in the control group not only complicate efforts to isolate the effects of the training program but also demonstrate that the psychosocial responses of young participants are multidimensional and sensitive to environmental influences. Therefore, monitoring social and environmental variables more thoroughly and controlling for them whenever possible in future studies is critical to enhancing the reliability of the findings and enabling a clearer assessment of the psychosocial effects of sport interventions (Table 3).

**Table 1:** Traning program.

**Week**	**Day**	**Activity description**	**Duration (min)**	**Intensity**
Week 1	Monday	Warm-up (20), running with direction and speed changes (15), football game (20), cool down (5)	60	Moderate
	Wednesday	Technical warm-up with ball (20), shooting techniques (inside, instep, outside foot) (35), cool down (5)	60	Moderate–high
	Friday	Warm-up (20), shooting practice (25), tactical game (15)	60	High
Week 2	Monday	Warm-up (20), paired running from different positions (15), football game (20), cool down (5)	60	Moderate
	Wednesday	Technical warm-up with ball (20), control drills (sole, top, inside, outside, knee, chest) (35), cool down (5)	60	Moderate
	Friday	Warm-up (20), coordination exercises (20), tactical game (20)	60	Moderate–high
Week 3	Monday	Warm-up (20), running focusing on speed and direction (15), football game (20), cool down (5)	60	Moderate
	Wednesday	Ball warm-up (20), dribbling (inside, outside, instep) (35), cool down (5)	60	Moderate–high
	Friday	Warm-up (20), shooting practice (25), tactical game (15)	60	High
Week 4	Monday	Warm-up (20), attacking runs (territory capture) (15), football game (20), cool down (5)	60	High
	Wednesday	Ball warm-up (20), defensive techniques (blocking, marking) (35), cool down (5)	60	Moderate–high
	Friday	Warm-up (20), coordination (20), tactical game (20)	60	Moderate
Week 5	Monday	Warm-up (20), confined-space chases (15), football game (20), cool down (5)	60	High
	Wednesday	Ball warm-up (20), dribbling and feints (35), cool down (5)	60	Moderate–high
	Friday	Warm-up (20), rhythmic gymnastics (20), educational game (20)	60	Low–moderate
Week 6	Monday	Warm-up (20), group running (speed changes) (20), football game (15), cool down (5)	60	Moderate
	Wednesday	Ball warm-up (20), off-ball deception movements (35), cool down (5)	60	Moderate–high
	Friday	Warm-up (20), rhythm and coordination with ball (35), cool down (5)	60	Moderate
Week 7	Monday	Warm-up (20), full-turn group runs (20), football game (15), cool down (5)	60	Moderate–high
	Wednesday	Ball warm-up (20), throw-in/corner kick practice (35), cool down (5)	60	Moderate
	Friday	Warm-up (20), rhythmic gymnastics (20), flexibility (20)	60	Low
Week 8	Monday	Warm-up (20), inverse running in 2 groups (20), football game (15), cool down (5)	60	Moderate
	Wednesday	Ball warm-up (20), set play exercises (35), cool down (5)	60	Moderate–high
	Friday	Warm-up (20), inter-group tournament matches (35), cool down (5)	60	High
Week 9	Monday	Warm-up (20), paired running (15), football game (20), cool down (5)	60	Moderate
	Wednesday	Ball warm-up (20), control exercises with various body parts (35), cool down (5)	60	Moderate
	Friday	Warm-up (20), coordination (20), tactical game (20)	60	Moderate–high
Week 10	Monday	Warm-up (20), attacking runs (territory capture) (15), football game (20), cool down (5)	60	High
	Wednesday	Ball warm-up (20), blocking and marking in different positions (35), cool down (5)	60	Moderate–high
	Friday	Warm-up (20), coordination (20), tactical game (20)	60	Moderate
Week 11	Monday	Warm-up (20), group running (speed changes) (20), football game (15), cool down (5)	60	Moderate
	Wednesday	Ball warm-up (20), off-ball deception movements (35), cool down (5)	60	Moderate–high
	Friday	Warm-up (20), rhythm and coordination with ball (35), cool down (5)	60	Moderate
Week 12	Monday	Warm-up (20), inverse running in 2 groups (20), football game (15), cool down (5)	60	Moderate
	Wednesday	Ball warm-up (20), set play exercises (35), cool down (5)	60	Moderate–high
	Friday	Warm-up (20), inter-group tournament matches (35), cool down (5)	60	High

**Table 2:** Demographic information of participants.

**Variables**	**Age**	**Height**	**Weight**
EG	13.2 ± 0.6	156.4 ± 6.1	46.5 ± 6.3
CG	13.0 ± 0.7	154.2 ± 5.8	48.1 ± 5.9

**Table 3:** Intra- and inter-group comparison of the exercise and control groups before and after training.

		**EG**					**CG**					**P_3_**	**np^2^**
**Scales**	**Test**	**Mean**	**S.D**.	**%**	***d***	**P**_1_	**Mean**	**S.D**	**%**	***d***	**P**_2_		
VTS	Pre	30.70	11.52	−2.11	0.056	0.832	39.70	17.90	−0.62	0.015	0.953	*P =* 0.939	0.000
	Post	30.05	11.48				39.45	13.83					
PRS	Pre	59.65	12.95	−14.50	0.654	0.035	50.15	14.05	−0.49	0.000	0.955	*P* = 0.153	0.053
	Post	51.00	13.49				49.90	9.44					
SAAS	Pre	46.10	10.77	1.19	0.046	0.839	43.50	12.09	−22.98	0.822	0.016	*P* = 0.029	0.120
	Post	46.65	12.62				33.50	12.22					

VTS, Violence Tendency Scale in Team Sports; PRS, Psychological Resilience Scale; SAAS, Social Appearance Anxiety Scale P_1_, EG group significance level; P_2_, Control group significance level; P_3_, Intergroup comparison significance level. *d*, Cohen d effect size; np2, partial eta squared.

## 4 Discussion

The present study was conceived with the objective of examining the psychological resilience responses of young athletes. The present study investigates the effects of an exercise programme on violent tendencies, anxiety, and psychological resilience. The findings indicate the emergence of disparate psychological tendencies between the exercise and control groups. Despite the absence of statistically significant changes in the Violence Tendency Scale (VTS) in either group, psychological resilience, as measured by the Psychological Resilience Scale (PRS), exhibited a significant decrease in the exercise group, while the Sports Anxiety and Aggression Scale (SAAS) revealed divergent tendencies between the groups. The study's findings suggest the presence of divergent psychological and perceptual tendencies within the exercise and control groups. The Violence Tendency Scale (VTS) results indicate a decrease in both the exercise and control groups. However, these changes were not statistically significant (*p* > 0.05), suggesting that the exercise programme did not directly impact violence tendencies. In Psychological Resilience Scale (PRS) assessments, a significant decrease was observed in the exercise group, and a moderate effect size was determined (*p* = 0.035; ηp^2^ = 0.053), while no significant change was observed in the control group (*p* = 0.955). This finding indicates that the exercise programme may temporarily reduce participants' subjective perception of improvement, which is likely related to the physical load and fatigue effects of training. The Sport Anxiety and Aggression Scale (SAAS) results demonstrated a non-significant increase in the exercise group and a significant decrease in the control group (*p* < 0.05; ηp^2^ = 0.120). These discrepancies suggest that the impact of the exercise programme on anxiety and aggression may be multifaceted and contingent on individual perceptions. The findings indicate that regular exercise programmes may exert a positive or negative effect on specific psychological variables. Furthermore, factors such as subjective improvement, anxiety, and aggression are influenced by individual experiences and perceptions.

Our findings indicate that 12 weeks of basic football training did not significantly alter violence tendency (VTS) or social appearance anxiety (SAAS) scores in children aged 12–14, while psychological resilience (PRS) showed a moderate decrease in the experimental group. This pattern aligns with previous research suggesting that football and similar team sports can impose temporary psychological and physical stress, especially during early stages of structured training (Tassi et al., 2023). Psychological resilience and exercise motivation significantly promote adolescents‘ sports participation, with exercise motivation playing a significant mediating role across genders and demographics (Hu et al., 2025). Participation in sports and physical activities contributes to psychological resilience among students by enhancing positive mental health, reducing negative emotions, and mitigating depression and anxiety (Husain et al., 2024). Increased sports participation is positively associated with increased resilience in children and adolescents, regardless of gender or school grade (Sheng et al., 2024). The observed decline in PRS may reflect training-induced fatigue, adaptation stress, or the challenge of coping with new physical and cognitive demands, consistent with reports that intensive or small-sided football programs can challenge young athletes' coping capacities even as they promote cardiovascular and musculoskeletal health (Krustrup et al., 2016; Demeke and Beyene, 2024).

These findings highlight the critical roles of training load, recovery, and individualized support in shaping young athletes' psychological responses. The temporary decrease in PRS in the exercise group likely reflects training-induced fatigue and adaptation stress. Carefully managing training intensity, volume, and progression is essential to prevent excessive stress and optimize both physical and psychological outcomes. Structured recovery, including active rest, sleep, and downtime, helps mitigate fatigue and maintain motivation. Differential responses in SAAS suggest that individual differences and environmental support moderate the effects of training, emphasizing the importance of personalized guidance. Overall, youth sports programs should integrate balanced training loads, recovery strategies, and individualized support to promote performance, resilience, and holistic development.

Although we did not observe significant improvements in social behavior or aggression, previous studies indicate that regular football participation can enhance social skills, reduce aggression, and foster self-awareness from preschool age through adolescence (Trajković et al., 2020; Mei, 2024). It is possible that the short duration and moderate intensity of our 12-week program limited the manifestation of such benefits, suggesting that longer-term, higher-frequency interventions may be necessary to observe measurable changes in social outcomes. Similarly, while football is reported to support cognitive functions and psychological skills such as resilience and motivation (Mao et al., 2024; Demeke and Beyene, 2024), our results imply that temporary decreases in perceived resilience may occur before longer-term adaptive improvements, highlighting the need for structured recovery periods and stress management strategies during training. Furthermore, the differential effects observed in PRS and SAAS suggest that psychosocial outcomes may respond differently to physical training depending on baseline psychological characteristics, individual differences, and environmental factors, such as family support or peer relationships (Delfin et al., 2023). This is consistent with literature emphasizing that sports participation can be both a source of stress and a resource for coping, and that monitoring and supporting young athletes' mental well-being is essential for maximizing the benefits of training (Ahmed, 2019; Erdogan and Koçal, 2024).

In summary, while our short-term football program did not significantly influence aggression or social anxiety, it temporarily affected psychological resilience, underscoring that youth sports interventions should consider training load, recovery, and individualized support to promote both performance and holistic psychosocial development (Murray et al., 2021). Future research should explore longer interventions with integrated recovery strategies, as well as potential moderating factors such as motivation, social support, and baseline psychological profiles, to better understand how team sports can optimize resilience, social skills, and overall well-being in children and adolescents (Sheng et al., 2024). Considering the current findings in conjunction with the existing literature, regular participation in football and other team sports appears to contribute not only to the enhancement of young athletes' physical fitness and musculoskeletal health but also to the development of their cognitive functions, psychological resilience, social competence, and emotional regulation (Graupensperger et al., 2021). Furthermore, when supported by social resources such as family and peer networks, engagement in sport has been shown to strengthen individuals' capacity to cope with stress and to mitigate the risk of adverse psychological outcomes, including depression and anxiety. Overall, these insights highlight that team sports serve as a valuable medium for the holistic development of young athletes and underscore the importance of integrating such activities into educational and training programmes to promote both performance and psychological well-being.

## 5 Conclusions

This study demonstrates that regular exercise programs have multifaceted effects on the psychological well-being of young athletes. While no significant change was observed in violence, different trends were observed in anxiety and aggression levels. However, significant changes in psychological resilience were observed in the exercise group, demonstrating that exercise programs can impact individuals' psychological adaptation processes in different ways. Consequently, regularly monitoring the psychological resilience and general mental state of young athletes and adapting exercise programs accordingly is crucial for supporting both performance and psychological well-being.

## 6 Limitations

This study has several limitations that should be considered when interpreting the findings. First, the sample was limited to children aged 12–14, which restricts the generalizability of the results to other age groups or populations engaged in different sports. Second, the intervention lasted 12 weeks, which, although longer than some short-term programs, may still be insufficient to capture long-term effects on psychosocial outcomes such as resilience and social anxiety. Third, all psychological variables—including perceived psychological resilience (PRS) and social appearance anxiety (SAAS)—were self-reported, which may have introduced subjective bias and influenced individual responses. Fourth, environmental factors such as school routines, family support, and peer interactions could not be fully controlled; this is reflected in the unexpected decrease in SAAS observed in the control group, suggesting that extraneous variables may have affected outcomes. Fifth, the relatively small sample size (*n* = 20 per group) may limit the statistical power to detect subtle changes in psychosocial variables. Sports during adolescence not only promote physical health, but also encourage the development of social skills, increased self-confidence and strengthened emotional resilience. Finally, the study focused on specific psychological outcomes, without evaluating broader physical, cognitive, or academic effects of football training. These limitations highlight the need for future research with larger, more diverse samples, longer intervention periods, multi-method assessments, and the inclusion of additional outcome measures to provide a more comprehensive understanding of the effects of youth football participation.

## 7 Recommendations

Young athletes' psychological state and anxiety levels should be regularly monitored during exercise programs.

Additional rest and recovery strategies to support psychological resilience (PRS) should be incorporated into programs.

Exercise duration and intensity should be adapted according to individual tolerance and physical capacity.

Psychological skills training and stress management techniques should be implemented to manage athletes' anxiety and aggression levels.

Coaches should personalize programs considering individual differences and plan supportive interventions when necessary.

Future studies should investigate long-term psychological effects across different age groups and sports, and the findings should be reflected in program design.

The observed temporary decrease in psychological resilience (PRS) suggests that children's recovery and stress levels should be closely monitored, and adequate rest and recovery strategies should be incorporated into training programs.

Individualized adjustments in training intensity and psychological support may help manage anxiety and aggression, optimizing both performance and psychosocial development in young athletes.

## Data Availability

The datasets presented in this study can be found in online repositories. The names of the repository/repositories and accession number(s) can be found in the article/supplementary material.
